# Systematic Literature Review of Barriers and Enablers to Implementing Food Informatics Technologies: Unlocking Agri-Food Chain Innovation

**DOI:** 10.3390/foods13213349

**Published:** 2024-10-22

**Authors:** William Alejandro Orjuela-Garzon, Angélica Sandoval-Aldana, Jonh Jairo Mendez-Arteaga

**Affiliations:** 1 Grupo de Investigación Bioecono, Inntegra SAS, Ibagué 730001, Colombia; 2 Grupo de Investigación Bioecono, Facultad de Ingeniería Agronómica, Universidad del Tolima, Ibagué 730001, Colombia; apsandovala@ut.edu.co; 3 Grupo de Investigación en Productos Naturales (GIPRONUT), Departamento de Química, Facultad de Ciencias, Universidad del Tolima, Ibagué 730001, Colombia; jmendez@ut.edu.co

**Keywords:** adoption, agri-chain, facilitators, food computing, inhibitors, Internet of food, transfer

## Abstract

Access to food products is becoming more and more complex due to population growth, climate change, political and economic instability, disruptions in the global value chain, as well as changes in consumption dynamics and food insecurity. Therefore, agri-food chains face increasingly greater challenges in responding to these dynamics, where the digitalization of agri-food systems has become an innovative alternative. However, efforts to adopt and use the technologies of the fourth industrial revolution (precision agriculture, smart agriculture, the Industrial Internet of Things, and the Internet of Food, among others) are still a challenge to improve efficiency in the links of production (cultivation), processing (food production), and final consumption, from the perspective of the implementation of Food Informatics technologies that improve traceability, authenticity, consumer confidence, and reduce fraud. This systematic literature review proposes the identification of barriers and enablers for the implementation of Food Informatics technologies in the links of the agri-food chain. The PRISMA methodology was implemented for the identification, screening, eligibility, and inclusion of articles from the Scopus and Clarivate databases. A total of 206 records were included in the in-depth analysis, through which a total of 34 barriers to the adoption of Food Informatics technologies (13 for the production link, 12 for the processing link, and 9 for the marketing link) and a total of 27 enablers (8 for the production link, 11 for the processing link, and 8 for the marketing link) were identified. Among the barriers analogous to the three links analyzed are privacy and information security and high investment and maintenance costs, while the analogous enablers are mainly government support.

## 1. Introduction

The world population is expected to exceed the threshold of nine billion people by 2050 [1], so the adoption of “Food Informatics” technologies is shown as a solution to the need to increase the production of more and better food [2] and overcoming the challenges of food security, food safety, sustainable development, and health [3]. Some research shows how data captured from smart agriculture can optimize yield with minimum inputs, reduce environmental pollution [4], and improve the market prices of agricultural products through decision-making on crop state and quality [5]. Likewise, applying technologies such as Blockchain facilitates transparency between farmers, markets, sellers, logistics, and consumers, combating fraud and generating trust throughout the agri-food chain [6].

It is clear that adopting this set of technologies throughout the agri-food chain is one of the determining factors for improving productivity and competitiveness [2] and facilitating data capture, interoperability, transmission, processing, and analysis. The complexity of actors that participate in the production chains, such as farmers, food processors, and marketers, besides the challenges they permanently face in the development of their activities—especially those associated with technology, human talent, and market changes—generates a permanent barrier to achieving the transformation and optimization of their activities.

At a global level, there are several examples of the use of data to improve efficiency in cultivation. Among these, the automation of robots and the increase in food quality based on artificial intelligence are highlighted (e.g., CGIAR Platform for Big Data in Agriculture, AgBioData consortium, and Global Open Data for Agriculture and Nutrition [GODAN]) [7]. However, even in the regional reality, there is a gap in the implementation of these technologies framed in three major causes: (1) the use and adoption of these types of emerging technologies [8,9], (2) the poor articulation and trust between the links in the chain for capturing and exchanging data under different standards and formats [10,11,12,13,14,15], and (3) the development of solutions fragmented per link that do not contemplate the bidirectional interoperability of data and decision-making under a farm-to-fork model [16,17,18]. These causes are even more relevant in contexts such as Latin America, where the economic dependence on agri-food activities is the basis of the livelihood of many peasant families.

At an international level, progress is being made in developing agri-food data repositories that allow, under the “open” logic, the exploitation and use of the same by the different links. This is the case with the Global Open Data for Agriculture and Nutrition (GODAN) (more information at http://aims.fao.org/godan_open_data, accessed on 19 March 2024), as well as the Internet of Food and Farm (IOF2020) (more information at https://www.iof2020.eu/, accessed on 17 May 2024) Project of the Horizon 2020 program of the European Commission, which seeks to develop a more sustainable food chain by using open data obtained through the Internet of Things (IoT) via a data ecosystem. Finally, the “Future Internet for Safe and Healthy Food from Farm to Fork” Project (some case studies of this project are available at https://kyklos40project.eu/, accessed on 19 March 2024) developed by the European Union agrees on the importance of developing a robust Internet infrastructure and improving real-time data processing from the food chain [19].

The Internet of Food provides new opportunities and has meant a crucial change in the digitalization of the agroindustry world. However, there are still critical gaps in harmonizing data generation, treatment, and processing derived from the different links of which they are composed and that permit the use of data analytics techniques to facilitate decision-making through the identification of patterns, trends, and factors that, in turn, allow the improvement of the agri-food chain performance in the departments and the country. Accordingly, the questions sought to be answered through this literature review are: What are the barriers (limitations) and enablers (drivers) for implementing technologies associated with Food Informatics in agri-food chains? What are the analogous barriers (limitations) and enablers (drivers) for implementing technologies associated with Food Informatics from a global view of the chain?

This study reviews relevant cases about the barriers and enablers to implementing Food Informatics technologies to address the research question: unlocking agri-food chain innovation. Moreover, by applying a comprehensive approach to the entire chain instead of an individual approach to each of its links, this work will contribute to strengthening the body of literature on barriers and enablers to implementing Food Informatics technologies in agri-food chains.

The paper is structured as follows: Section 2 provides the theoretical background of the research, Section 3 presents the methodology applied in this paper, Section 4 provides the analysis of the results at various levels, including metrics, knowledge structures, and identified barriers and enablers for the production, processing, and marketing links, and Section 5 discusses the results obtained. Finally, Section 6 includes the conclusions from this study and recommendations for future research.

## 2. Theoretical Background

Agri-food chains, understood as complex networks that connect agricultural systems with final consumers through production, processing, storage, and distribution processes [20], are highly susceptible to events such as climate change, market readjustments, armed conflicts, ecological disasters, changes in consumption habits, and food policies [21]. A simplified view of the agri-food chain shows three main links: production (cultivation), processing (food production), and final consumption [22].

**Figure 1:**
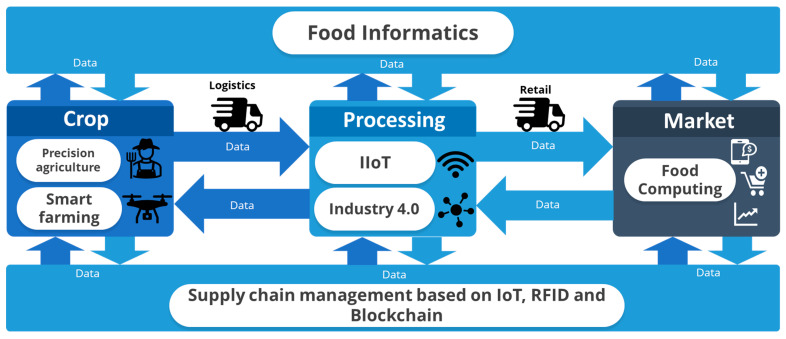
Bidirectional model of data generation and use in agri-food chains. Elaborated upon by the authors based on [25].

The first link in the chain is food production or cultivation, where multiple interrelated variables such as soil, climates, and plants must be analyzed and optimized. In this case, precision agriculture, or precision farming, allows for the monitoring, controlling, and treating of crops and land to reduce their spatial and temporal variability, facilitating appropriate treatments in time and place [23]. This concept gives rise to smart agriculture, which focuses on using information and communication technologies that increase production quality and quantity while improving the efficiency of human intervention [24]. This is achieved through the implementation of weather stations, precision irrigation, sensors (soil, water, light, humidity, and temperature), software, location systems (GPS and satellite), communication systems (Wi-Fi, Lora, Bluetooth, Zigbee, and LoraWan, among others), and robots (drones, among others) (see Figure 1).

For the processing link, where raw materials obtained in production are taken and food processing processes are carried out, these transformation processes contemplate the use of equipment and control parameters such as temperature, time, pH, humidity, and pressure, among others. In this case, the Industrial IoT (IIoT) emerges, allowing the control of entire processing plants through interconnected sensors [26]. This approach, also known as industry 4.0, allows for the increase of the production of final goods through automation and predictive maintenance, as well as the optimization of the decision-making process through the management of information in real-time [27] (see Figure 1).

Regarding the consumption link, where the products elaborated upon directly satisfy client needs, different studies focus on capturing perceptions and the recognition, recommendation, and monitoring of food. Therefore, the concept of Food Computing is a research approach focused on the consumer who supports his/her purchasing decisions based especially on social networks (Facebook, Twitter, Instagram, and YouTube Foursquare, among others), IoT, and web media [28], and the data obtained are analyzed through text-mining techniques and the analysis and processing of natural language or opinion mining, enabling the understanding of the habits, the perception of health that consumers have, as well as how recommendable certain foods are (see Figure 1).

Finally, as a transversal element between the three links described above, there is the logistics and supply of food, which facilitates the flow of ingredients and finished products under hygiene, temperature, and storage conditions. Hence, integrating IoT, Radio-frequency identification (RFID), and Blockchain technologies allows for the monitoring of quality variables and the improvement of food tracking and its traceability [29,30] (see Figure 1).

The specialized literature integrates the different perspectives mentioned above from the concepts of Computer Science under the notion of Food Informatics, which, for this research proposal, will be understood as the collection, preparation, analysis, and intelligent use of data from agriculture, supply chains, food processing, and consumers for the extraction of knowledge and the development of intelligent analysis that allows for the optimization of agro-chains based on data analytics [25].

This model of the integration of various data sources is supported by (1) efficient technology adoption processes that enable the development and optimization of decision-making processes [31] in the different links from a bidirectional perspective and (2) models of articulation and trust between the links to promote data exchange.

Regarding the first, it cannot simply be assumed that just because a technology exists, it is known and used effectively [32], since the more complex the technology is, the higher the level of cooperation required between the parties [33,34]. Under this logic, specialized literature has been analyzing the factors that inhibit or enhance the adoption and use process under different technology acceptance models, such as the Technology Acceptance Model (TAM), which has been proposed to understand the drivers of human behavior [35]. The analysis of the decision-making factors allows understanding adoption profiles and the special requirements in each chain link, facilitating the development of strategies and policies so that decision-makers can accelerate the process.

Regarding the second element, the high complexity of the Food Informatics model requires the participation of all interested parties, who have different roles in the data value chain [24], as well as data exchange, collaboration, acquisition, and management frameworks, forcing a change in the data ecosystem [36]. As stated before, technologies related to Food Informatics are changing the way value is created for small and medium-sized companies [37], where data becomes another asset that must be managed through business models that encourage those involved to collect and exchange data in an accessible and interoperable way that allows for the reuse of these, creating and capturing value to develop advanced analytics processes, and that does not only involve one link, but, on the contrary, has a complete view of the multiple data sources.

Transparency and traceability in the food industry have become central elements for consumers and companies. In the case of consumers, there is a need for more information, and regarding the processors and manufacturing companies, they must mitigate food challenges and reduce costs and fraud to improve their impact on the market [38]. Under this logic, in Table 1, the technologies associated with the Food Informatics model and its potential applications in the different links of the chain are shown.

Regarding the adoption processes of Food Informatics technologies, different theoretical approaches have been developed based on models that have their foundation in the discipline of sociology. Among these, the case of the study carried out by Rogers (1962) is highlighted [39]. Each model postulates variables that attempt to explain the principles on which behaviors are established around the decisions of adopting (or not) a technology from the exploiter perspective. These include the theory of reasoned action (TRA), the theory of planned behavior (TPB), the theory of diffusion of innovation (IDT or DOI), the unified theory of acceptance and use of technology (UTAUT), and the technology acceptance model (TAM) (Tohidyan and Rezaei-Moghaddam, 2017) [40].

**Table 1:** Technologies associated with the Food Informatics ecosystem.

Link	Technology	Description/Application	Support References
Production—Processing and Marketing	Artificial Intelligence (AI)	It simulates human intelligence, allowing information to be processed and learned, and problems to be solved optimally. AI components are embedded in applications for climate management, waste control, nutrition management, disease detection and treatment, demand projection, quality management, inventory control, consumer analysis, and food fraud detection.	[41,42,43,44,45,46]
Production	Drones	Uncrewed aerial vehicles. These can be adapted with different technologies to monitor activities, product application, inspection, topography, and cartography. These data are reported and stored for decision-making.	[42]
Production—Processing and Marketing	IoT	These are systems of interconnected sensors that communicate and interact with each other, allowing for the capture of information from actuators. These have the potential to be applied in different links of the agri-food chains due to their impact on sustainability, energy consumption, manufacturing costs, security, supply chain tracking, marketing, and consumer experience.	[47,48]
Processing	IIoT	These are sensors, devices, and industrial machines interconnected to capture information and share it in real-time. Their applications are primarily found in the processing link because they allow for the optimization of production processes based on the real-time monitoring of process variables, quality control, traceability, logistics, and inventory management. This technology facilitates the implementation of smart factories.	[49,50]
Production—Processing and Marketing	Blockchain	Blockchain can be defined as “a decentralized and distributed data recording system in which transactions are recorded and added in chronological order to create permanent and tamper-proof records.” This technology is applicable throughout the agri-food chain, facilitating the traceability, quality verification, and certification of origin processes. It also increases food safety, brand reputation, and end-consumer satisfaction.	[51,52,53]
Production—Processing and Marketing	Cloud Computing	Virtual storage systems accessed through the Internet; these are only accessed via an Internet connection and avoid the use of local applications such as physical servers and computers. This service model can be implemented to apply technologies such as IoT, Blockchain, and AI, among others.	[54,55,56,57]
Production—Processing and Marketing	Edge Computing	These are data processing models that allow for increased response times because they act as a cloud closer to local devices and end users. This technology is applied in the production, processing, and marketing links since it supports others, such as machine learning, IoT, and Blockchain.	[58,59,60]
Production—Processing and Marketing	RFID	This is a radio-frequency identification system that allows for the tracking of products in real-time, inventory management, quality control, and maintenance processes, increasing transparency.	[61,62]
Production—Processing and Marketing	Robotics–Cobots	This is the application of robots designed to interact directly with humans in shared spaces, allowing for the more efficient development of tasks, reduction of risks, and improvement of process automation. These robots are mainly used in the production and processing links; however, their use extends to the marketing link.	[63,64,65]
Production—Processing and Marketing	Digital Twins	These are virtual representations of physical entities that are permanently updated through data to generate dynamics that allow decisions to be made in the real world. Their applications range from cultivation and processing to logistics and food packaging.	[48,66,67]

Authors such as [68,69] have established reference frameworks to improve the understanding of the adoption processes of Food Informatics technologies, especially Blockchain, identifying three basic stages: (1) initiation, where the need is recognized, and planning and a first pilot are carried out; (2) the adoption decision stage, where the technology is adopted, and the necessary resources are defined; and (3) the implementation process, which is divided into the implementation process itself and the process of integration with the business, and connecting it with other existing applications. It is clear that there are barriers and enablers that positively and negatively impact this process.

## 3. Materials and Methods

The PRISMA (Preferred Reporting Items for Systematic Reviews and Meta-Analyses) methodology proposed by Moher et al. (2009) [70] was used to develop the systematic literature review process. This methodology proposes four stages: (1) identification, (2) screening, (3) eligibility, and (4) inclusion. For the identification stage, keywords were used according to the link in the food agro-chain that was being analyzed (see Table 2, Table 3 and Table 4), and a search for records was carried out in two databases, Scopus and Web of Science (WoS). All available collections [the Core Collection, which covers the Sciences, Social Sciences, and Arts and Humanities indexes, as well as the Proceedings of both Sciences and Social Sciences and Humanities] and complementary databases [Medline, Scielo and Korean Citation Index, BIOSIS Previews, and FSTA—The Food Science Resource] were used for the search, and were explored in February 2024 using structured search equations based on Boolean operators and field codes (title–abstract–keywords) for the last 10 years [69,71] (see Table 5, Table 6 and Table 7). The process of constructing the search equations for each link of analysis is shown below.

**Figure 2:**
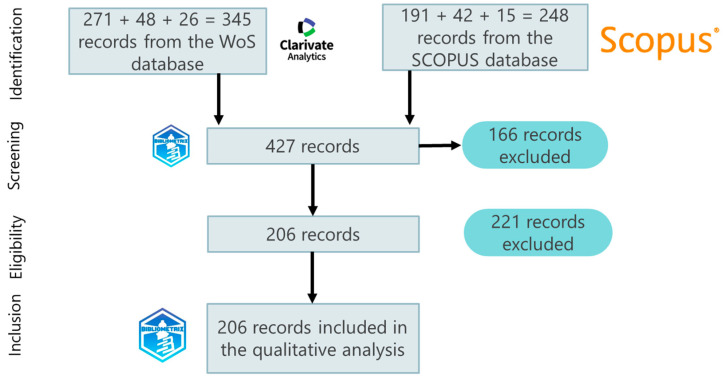
PRISMA methodology. Source: adapted from Moher et al. (2009) [70].

A total of 593 records were obtained for the identification stage, coming from the two databases consulted and the summation of the results of the three links of the agri-food chain analyzed (production, processing, and marketing). For the second stage, i.e., screening, duplicate records were eliminated using the method described by Caputo and Kargina (2022) [72] in the Bibliometrix 4.1 software [73] yielding a total of 427 unique records. For the eligibility stage, two exclusion criteria were established: articles that do not mention barriers or inhibitors to implementing Food Informatics technologies, and articles that do not address case studies in the agri-food sector. Under these criteria, 221 documents were excluded after the in-depth review. Finally, in the inclusion stage, the number of documents chosen for qualitative and quantitative analysis was 206 records. Figure 2 graphically indicates the procedure for the preferred information elements for systematic review and meta-analysis.

## 4. Results

The Bibliometrix 4.1 software developed by [59] was used to analyze the results, which facilitates the quantitative analysis of bibliographic records related to a specific area of knowledge from different bibliometric techniques and is supported by the R programming language. In general terms, 206 documents were obtained with an interannual growth rate of 33.51% and an international co-authorship of 28.64%. In addition, these documents have been published in more than 152 different journals by more than 760 different authors.

The analysis was conducted through two levels of metrics and one level of knowledge structures. The first was mainly related to the annual scientific production, the primary sources, authors, and documents, and the second was focused on the identification of the conceptual, intellectual, and social structures of the studied subject.

### 4.1. Level of Metrics

**Figure 3:**
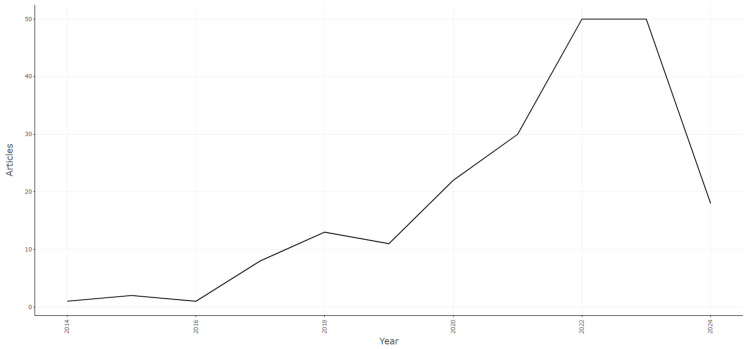
Scientific production (number of articles published) from 2014 to 2024. Source: elaborated upon by the authors based on the Bibliometrix 4.1 software [73].

The scientific production related to the barriers to implementing Food Informatics technologies shows a growing interest, with a total of 206 documents for the 2014–2024 period, with the years 2022 and 2023 being the ones with the highest production, with 50 documents each. Likewise, it is evident that since 2019, the interest in this research topic has increased from 11 documents to 22 in 2020, a rise of 100%. Regarding 2024, there is a significant decrease in scientific production, mainly due to the indexing status of documents at the date of consultation in the respective databases (see Figure 3).

**Figure 4:**
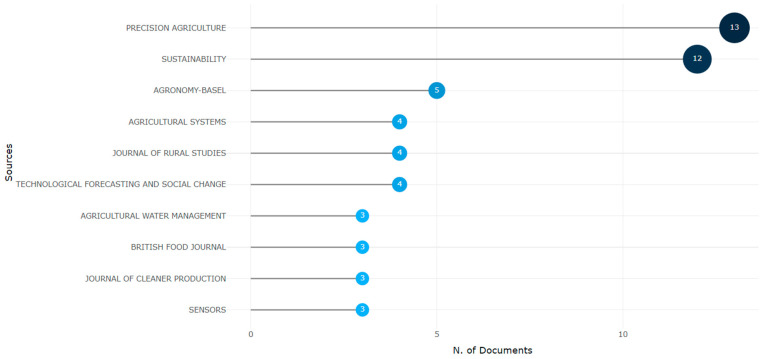
Most cited sources. Source: elaborated upon by the authors based on the Bibliometrix 4.1 software [73].

Among the most important journals related to the research topic, Precision Agriculture with 13 articles, Sustainability with 12 articles, and Agronomy with 5 documents are highlighted (see Figure 4). A thorough review shows that this new knowledge base associated with the Food Informatics topic is supported by high-impact journals such as Computers and Electronics in Agriculture, a journal with a long history in the areas of crop science and applied computer science, among others, and with an H index of 168. Likewise, Precision Agriculture appears again, focused on developing innovations in information systems for agriculture and agri-food chains; this journal has an H index of 86.

Among the most cited articles, “Blockchain technology adoption, architecture, and sustainable agri-food supply chains” stands out. This research was developed by Samant Saurabh of the Indian Institute of Management and has more than 208 citations. Overall, this publication answers the following three questions related to the object of this research.

- Question 1: What factors influence supply chain actors to adopt Blockchain technology, and how can these factors streamline information systems architecture?
- Question 2: How can information systems architecture make the supply chain inclusive and increase support for value creation for actors?
- Question 3: How can information systems architecture ensure food quality and safety and impact sustainable supply chain practices?

**Figure 5:**
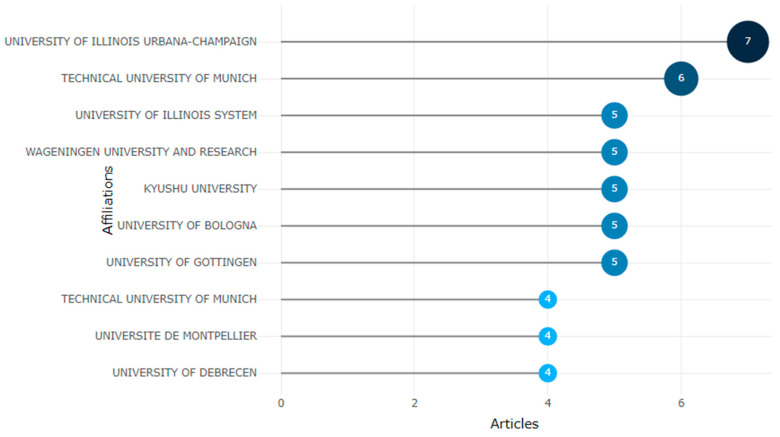
Most relevant affiliations. Source: elaborated upon by the authors based on the Bibliometrix 4.1 software [73].

Among the institutions hosting research (affiliations) in this field of knowledge, mainly German universities stand out, such as the Technical University of Munich with six publications and the University of Wageningen with five publications. However, the leading institutions in publication are the University of Illinois URBANA-CHAMPAIGN, with seven, followed by another American university, the University of ILLINOIS SYSTEM, with five publications. Likewise, European universities such as the University of Bologna and the University of Montpellier also appear (Figure 5).

The countries that concentrate the publications in the study area are Italy, the United States, and India, with 20, 20, and 18 publications, respectively. Brazil appears in the top eight, with six publications at the Latin American level. Colombia is ranked 34th with only one publication.

### 4.2. Level of Knowledge Structures

**Figure 6:**
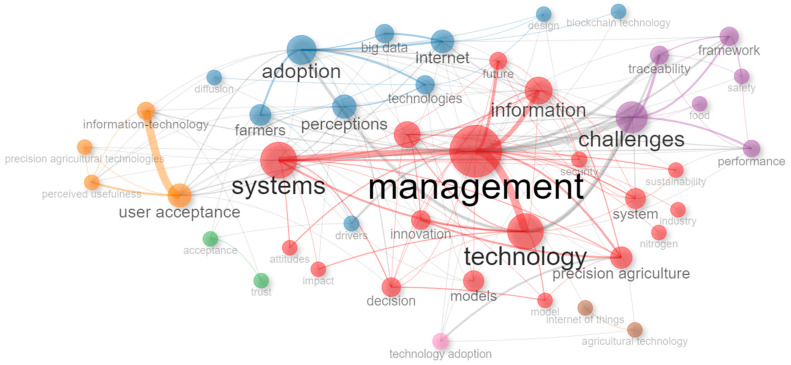
Theoretical structure. Source: elaborated upon by the authors based on the Bibliometrix 4.1. software [73].

Using the ConceptualStructure function to perform multiple correspondence analysis (MCA) to draw a conceptual structure of the field and K-means grouping to identify groups of documents that express common concepts based on the occurrence of words between the records analyzed, four main groups or nodes were evident and can be observed in Figure 6.

The first is the red node, which is associated with research related to the management of information and decision systems; the second is the blue node, associated with adoption from the perspective of perceptions and drivers that influence it; the third is the purple node, oriented to the challenges, scalability, and performance of the adoption processes of Food Informatics technologies in agri-food chains; and finally, the fourth is the orange node, associated with technology adoption and acceptance models, guided by theoretical models such as the Unified Theory of Acceptance and Use of Technology (UTAUT) [74], the Technology Acceptance Model (TAM) [75], and the Theory of Reasoned Action (TRA) [76].

**Figure 7:**
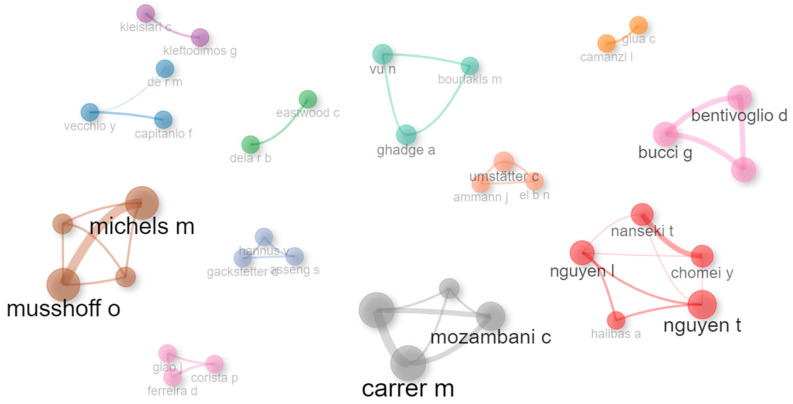
Author collaboration network. Source: elaborated upon by the authors based on the Bibliometrix 4.1 software [73].

Regarding the interaction between authors, the green cluster stands out, comprised mainly of three authors, Nam Vu, Abhijeet Ghadge, and Michael Bourlakis, all linked to the Centre for Logistics and Supply Chain Management in the School of Management at Cranfield University in the United Kingdom. These authors have focused their research on the implementation processes of Blockchain technologies in agri-food chains. Then, a brown cluster can be observed, led by authors such as Oliver Musshoff and Marius Michels, both linked to the Georg-August University of Göttingen in Germany, Department of Agricultural Economics and Rural Development. They have focused their research on analyzing the determinants of adopting technologies such as drones, smartphones, the Internet, and mobile applications in agriculture, which can enhance small- and large-scale agriculture and help close the digital gap in this sector. Finally, a grey cluster stands out, led by Marcelo José Carrer and Carlos Iván Mozambani, both linked to the Department of Production Engineering of the Federal University of São Carlos in Brazil. Their research focuses on the analysis of the determinants of the adoption of precision agriculture technologies in agro-industrial chains through selectivity analysis for stochastic frontiers and meta-frontier production functions (see Figure 7).

**Figure 8:**
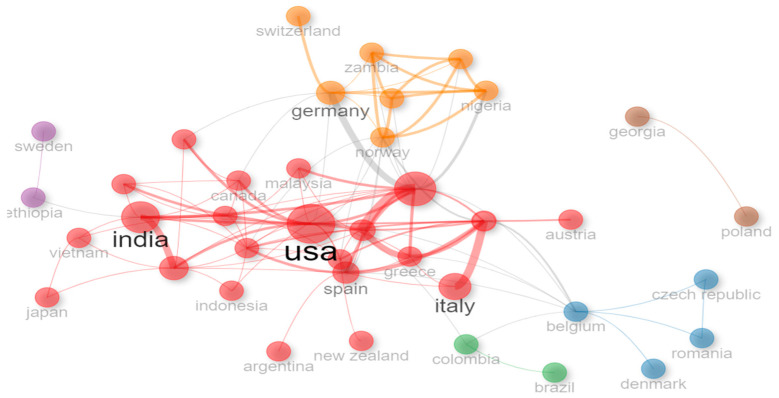
Country collaboration network. Source: elaborated upon by the authors based on the Bibliometrix 4.1 software [73].

Three main nodes are identified at the level of collaboration networks between countries (see Figure 8). The first one is red and is comprised of countries such as the United States, India, Spain, Italy, and the United Kingdom. This last country is the link to the yellow node, which includes Norway, Germany, Switzerland, Zambia, and Nigeria. Finally, a blue node stands out, comprised of Belgium, the Czech Republic, and Romania. This node is dynamic and presents interaction with the main node (red) and a green node that articulates Latin American countries such as Colombia and Brazil. Regarding Brazil, it is worth highlighting that its presence is strengthened because it links one of the most prominent authors in the production of knowledge regarding the adoption of technologies in the production link, i.e., Marcelo José Carrer.

### 4.3. Barriers and Enablers Identified for the Production Link

Table 8 shows the barriers identified for the production link. The adoption and use of Food Informatics technologies in the production link is one of the most predominant research approaches, especially due to the impact on productivity and cost reduction that smart farming, precision agriculture, agriculture 4.0, and digital agriculture technologies have. These have shown how different interconnected devices impact product quality and environmental sustainability and reduce the risks associated with cultivation, all based on access to information and knowledge by farmers.

Several barriers are identified in the literature for adopting Food Informatics technologies in the production link. It is evident that most research has focused predominantly on this link, as it has the lowest adoption percentage. Among the barriers, complexity is understood as a multidimensional factor that is associated with several factors, not only technical, but also social [77]. This technological complexity can be reduced if the farmer has the ability to experiment with the technology without incurring costs, evaluating the advantages or disadvantages, and, thus, assessing compatibility [77].

**Table 8:** Barriers identified for the production link.

Barrier	Description	Chain	Technology	Reference
Interoperability	This allows technologies to exchange data in a fluid manner.	Banana	Agriculture 4.0	[10,78]
Age	The chronological age of potential users influences their risk perception and usefulness levels, which is why many studies have focused on evaluating how young people or adults exhibit different patterns of technology adoption.	Coffee Pisciculture	Agriculture 4.0 Precision farming Smartphone Smart weeding Wireless sensor networks	[10,13,14,15,79,80,81,82,83,84,85,86,87,88,89,90,91,92,93,94,95,96,97,98]
Cost	The costs associated with the purchase, rental, and maintenance of technologies reduce or motivate their adoption.	Not mentioned	Agriculture 4.0 Smartphone	[10,12,13,79,90,93,94,96,99,100,101,102,103]
Complexity	It is related to the difficulty in understanding, controlling, and managing the technologies to be adopted.	Not mentioned	Agriculture 4.0	[4,10,13,77,104,105]
Privacy and information security	The privacy and security of information poses a risk for the parties involved, which is why it becomes a risk that farmers prefer not to take.	Not mentioned	Agriculture 4.0 Robotic Smart weeding	[10,11,12,13,14,15]
Data standards	How the organization is structured is a barrier, given that low data quality and interoperability become barriers to its reuse and comparability.	Not mentioned	Agriculture 4.0	[10,13]
Time consumption	The time commitment required for implementing and using technologies can limit their adoption.	Not mentioned	Agriculture 4.0	[10,13,80,98]
Compatibility	The adoption of technologies also depends on the ease with which they can be coupled with other technologies already implemented.	Not mentioned	Agriculture 4.0	[8,13,79,93,94,104,105,106]
Infrastructure	The physical infrastructure required for the implementation of technologies, both internal and external, limits their adoption and use.	Not mentioned	Agriculture 4.0 Precision agriculture	[12,101,104]
Attitude towards risk	The perception of risk, especially in terms of return on investment, limits the adoption of technologies.	Banana	Precision farming IoT	[4,8,13,78,80,93,94,103,104,107,108,109]
Relationship with extension agents	Technologies promoted by external agents require assertive communication and relationships between the parties to achieve their proper implementation.	Sugarcane Maize	Precision farming	[8,13,79,81,94,96,99,108,110,111]
Farm size	The size of the farm has been analyzed as a limitation in the adoption of technologies; farms of smaller size are especially less likely to adopt.	Grapes Coffee Maize	Satellite maps Smartphone Smart weeding	[4,13,14,80,81,82,83,84,90,92,94,95,96,103,108,111,112]
Connectivity	Technologies associated with Food Informatics require an Internet connection, so infrastructure is necessary in production areas.	Not mentioned	Information management system Agriculture 4.0 Smartphone	[10,90,100,104,113]
Job losses	Farmers associate the adoption of technologies with the loss of jobs, so they prefer to abstain from using them.	Not mentioned	Precision agriculture	[12]

The attitude towards risk is another determinant for technology adoption, mainly associated with the willingness to invest in new technologies, which is affected by the uncertain economic returns these investments can bring [78,104,107,109]. Regarding the farm size, it is evident that those farmers with larger farm sizes promote the use of technologies for production control. However, this is significantly reduced when organic practices are introduced [92]. On the other hand, other research contradicts these findings, stating that farm size does not impact the adoption of precision agriculture technologies [114]. Likewise, land ownership plays a role in the adoption process, since if the land is owned or leased, the willingness to make adjustments or adaptations required to implement the technology at the infrastructure level restricts the probability of adoption [83,102,115].

Age is one of the determining factors in adopting technologies in the production link, mainly because older producers are more conservative and less susceptible to taking risks when implementing technologies. Other research shows how younger producers are perceived as more open to implementing technologies and developing innovations [79,87]. Another of the factors identified in the literature is costs. It has been suggested that producers with high incomes are more likely to make investments than those with medium or lower incomes. Likewise, research shows that some highly technological crops require more significant investment and, thus, greater returns that promote the adoption of technologies [116].

Time consumption, also referred to in the literature as the relevance of work, is mainly associated with reducing workload or physical intensity when implementing Food Informatics technologies, mainly associated with robotization, where the physical load is substantially reduced in tasks [98]. In this sense, the compatibility associated with the technology is also a widely studied factor, mainly associated with perceived usefulness and ease of use. If the technology is incompatible with the current infrastructure, it can generate uncertainty, leading to low adoption [117]. On the other hand, if the technology is customized to fit end-user requirements, it is much more likely to be adopted [93].

Data security is a factor that has been less studied in the literature regarding the production link. However, it is essential to highlight that it is a key aspect when collecting data from farmers because it is necessary to guarantee transparency and generate trust among the other parties involved to achieve adoption processes [93]. Research conducted by [11] raises the need to define legal frameworks for the adoption of precision agriculture technologies, especially robots, due to legal aspects such as the operation and use of these devices, as well as privacy and the use of information collected by these devices, showing that there are still some gray areas in the current global legislature that could promote and facilitate the adoption and investment of this type of technology [11].

Infrastructure and connectivity are two closely related factors because, if these facilities or installations are not available, the deployment of some technologies is extremely complex and increases their costs because the farmer must cover the costs of deploying electric power, fiber optics, or Internet devices that allow data capture and processing [12]. Other research has focused on the effect of using mobile Internet as a solution to the low infrastructure and speed in rural areas, positively impacting the use of technologies in agriculture [118].

The relationship with the extension agent associated with support in the technology implementation processes plays a key role. Beyond the purchase or acquisition and installation of the technology, continuous support processes are required to motivate end users [94]. Finally, job losses have been analyzed from the perspective of the effects of process and procedure automation, which could put pressure on job security and the inclusion of young people in the next generation of farmers [12].

Table 9 shows the enablers identified for the production link. The enablers of technology adoption in the production link are associated with both internal and external elements: for example, on the one hand, the educational level and the skills required for a correct adoption process, and on the other hand, government support and ease of access to credit, among others. At a specific level, the relative advantage is mainly associated with the potential for value addition and efficiency in terms of business management that the incorporation of technologies can provide [107], i.e., the relative advantage is linked to the perception of profitability that the technology can generate on the crop, and a positive effect on the intensity of adoption is evident [97].

Associated with this, those farmers with few resources can access credit to acquire the technologies. Studies show that credit services increase adoption rates more with government subsidies [119]. Access to credit can be promoted through government policies such as programs aimed at promoting adoption processes, which play a key role in adopting technologies, mainly among young farmers who are more willing to participate in these programs [107,120]. The development of policies plays a vital role in encouraging the adoption of technologies by increasing the expected returns and reducing potential risks [121,122].

**Table 9:** Enablers identified for the production link.

Enabler	Description	Chain	Technology	Reference
Access to credit	Acquiring technologies through credit facilitates access to technology, since it allows current access with the commitment to pay for it in a certain period.	Sugarcane	Precision farming	[13,79,110]
Ease of use	Ease of use is an enabler studied through different technology acceptance models and is directly associated with the simplicity and intuition with which a user can use the technology. Complex technologies are less adoptable.	Not mentioned	Agriculture 4.0 Precision agriculture	[10,13,79,89,93,98]
Perceived usefulness	Ease of use is an enabler studied through different technology acceptance models and is associated with the degree to which a user believes that the technology will improve their performance or solve the identified problem.	Sugarcane	Artificial intelligence	[13,81,89,93,98,110]
Level of education	The educational level has been analyzed as an important variable in the adoption processes, considering that it can range from secondary to postgraduate levels.	Sugarcane Pisciculture Maize	Agriculture 4.0 Smartphone Smart weeding	[15,78,82,86,88,90,91,111,123,124]
Relative advantage	The level of efficiency or competitiveness that adopting a technology can provide is also a key element for technological adoption.	Not mentioned	Agriculture 4.0 IoT Precision agriculture	[4,8,10,13,15,93,97,103,105,106,107,125]
User training, skills, and experience	The skills and experience of the user facilitate the process of importing and using the technology.	Sugarcane	Agriculture 4.0 Smartphone	[10,12,81,82,89,94,96,100,108,110,125,126]
Relationship with neighbors	The possibility of sharing experiences and learning through associative networks or even neighbors generates confidence about using or not certain technologies.	Not mentioned	Fertilization technologies Wireless sensor networks	[4,15,89,95,127]
Government support	Policies associated with promoting and using technologies through programs or subsidies can facilitate the adoption process because they reduce acquisition, operation, maintenance, and training costs.	Not mentioned	Precision agriculture	[15,107,120,121,122]

Some barriers can act as enablers; this is the case with the level of education and skills of the user. It is especially evident that adoption rates can increase if the level of education of the farmer is higher. Furthermore, if the technology is advanced, specific learning and training processes are necessary [79]. Research shows that farmers with postgraduate education levels are more likely to adopt precision agriculture technology, especially at the master’s level [80,87]. Likewise, some technologies require training and practical learning processes to improve their ease of use and perceived usefulness [94].

As with the educational level, training, skills, and user experience can act as barriers or enablers [125]. However, this factor positively correlates with the adoption process and educational levels. In other words, farmers with more experience in the activity are more likely to adopt technologies [108]. Although empirical studies present evidence of the effect of experience on the adoption of technologies [119], it is also evident that younger farmers have less risk aversion and are more willing to learn about digital technologies such as precision agriculture [128]. Other authors have associated experience and skills with attitudes and beliefs that can be considered when making decisions about the adoption of technologies [82].

Regarding ease of use, a factor associated with technology acceptance models that has been widely analyzed in the literature, it is evident that demonstration and networking processes facilitate adoption processes in the agricultural sector. This factor is associated with perceived usefulness, which has a direct impact on ease of use, thus positively influencing the adoption of technologies; however, it shows an inverse (negative) relationship with the complexity factor, which acts as a barrier [93].

Other research shows evidence of how the social context can play a role in the motivation towards technology adoption, especially the relationship between neighbors, which could imply the generation of knowledge spillovers through demonstrations and visibility of the technology [123], reducing the cost of learning [95,129]. Likewise, some research has been aimed at demonstrating how agricultural cooperatives play a role in innovation processes, acting as intermediaries for the promotion of technology adoption [123]. This social influence derived from the opinions generated by other users is a determinant of technology adoption [4], as well as advertising through digital media [123]. Finally, regarding formal and informal sources for access to information, some research has shown that formal sources increase perceived utility and, therefore, adoption. In contrast, informal sources do not reduce perceived utility [130].

In general terms, the relationship with actors such as suppliers, consultants, research institutions, and early adopters generates a key benefit primarily related to exchanging information and creating knowledge [127].

### 4.4. Barriers and Enablers Identified for the Processing Link

Table 10 shows the barriers identified for the processing link. The agro-industrial landscape is evolving, and the use of technologies such as Blockchain, industrial IoT, robotization (cobots), and additive manufacturing, among others, allows for the improvement of product quality and value, as well as the optimization of processing (transformation) processes, creating competitive advantages [9,131]. These intelligent systems that use sensors enable monitoring, exchanging data even between languages and models that are not the same, achieving interoperability [132], and integrating models and resources that operate in a network to generate manufacturing environments and collective logistics [131], which allow for the implementation of the factories of the future, increasing the innovation potential.

This approach, understood as intelligent production, is divided into four parts of a life cycle: logistics, production, design, and marketing, impacting the nutritional quality of the products through human–machine integration and delivering to the end customer products that are much more adjusted to the requirements, mainly due to the impact that storage, transportation, and even packaging can have, affecting the health, safety, taste, and stability of food [48]. Adopting Food Informatics technologies in the processing link can improve the quality and safety of food that reaches the consumption link [9]. Despite this, in the agro-industrial chains, especially for the processing link, there is still a long way to go to reach these levels of automation.

Connectivity is a critical element when deploying technologies such as Blockchain in processing processes, so if connectivity is poor, the environment is not ideal for the operation of electronic devices [133]. Adopting technologies such as Blockchain requires the understanding and skills necessary to support all aspects of the technology, so limited technical skills and knowledge present a critical barrier to its adoption [133]. Small companies may not have enough understanding and experience to allow them to commit to the full implementation of Food Informatics technologies, which becomes a limitation for their adoption [9,139]. Likewise, research has shown that successful implementation processes of this type of technology have been achieved through a learning-by-doing process, through which the firm increases its digital skills [137].

Collaboration between the different links operating in the value chain is key not only for the joint implementation of technologies, but also for maintaining the transparency, trust, and traceability of data because a change in data chains can generate nonconformity and fraud [136]. Similarly, the collaborative and multidisciplinary role of the actors in the food industry is key to developing intelligent or smart food factories, which facilitate the development process of new and better products, as well as the optimization of inputs and benefits, guaranteeing quality and safety, reducing food waste, and tracking food from “farm to fork” [138].

The maturity and preparation level of the chain actors are mainly associated with the enabling conditions of the environment, i.e., support infrastructure, human capital, and organizational support can increase the availability of and reduce the effort to implement Food Informatics technologies [145]. On the other hand, the lack of knowledge of the real benefits also becomes a barrier to the adoption of technologies, mainly because, in some cases, it cannot be verified whether it will have a positive result on the process to be improved or if, on the contrary, it will generate many more problems [139,140]. This lack of knowledge of the benefits is associated, in some research, with not having heard or known about these technologies through collaborative networks [139].

At the technological level, the obstacles presented by Food Informatics technologies are oriented to the specific challenges of each technology. Likewise, the need for cost-effective and efficient technologies is challenging, as is the ability to convert these investments into added value for the Food Industry [138]. The literature shows that technologies such as Blockchain can add value to value chains through data and necessary information that must be exchanged transparently between the different links. Hence, authenticity needs to be guaranteed through protocols, standards, regulations, and national policies [133].

Data privacy is a priority aspect for processors because it allows for the generation of trust and reliability for the agri-food chain as a whole [134]. In this sense, the immutability of records and transactions can help producers, processors, and distributors to identify fraud. This element of data privacy is also linked to safeguarding the intellectual property of the actors involved and ensuring no security breaches [138].

Investment and maintenance costs, as well as the complexity and dedication of time and resources [9], become challenges for implementing Food Informatics technologies in the processing link [135]. Some research shows that investments in implementing Food Informatics technologies are perceived as unnecessary, not only because of the costs of the technology, but also because of the adjustments and other hidden costs of its maintenance and operation. However, other research shows how investment in technologies such as Blockchain can become an element that returns the investment quickly, reducing other costs [9].

In cases of resistance to change not only by employees, but also by the organization itself in the different phases of adoption, employees prefer to continue operating under regular routines and habits instead of adopting changes in their jobs to implement the technology [135]. Trust between firms is a key enabler for adopting Food Informatics technologies, especially when different organizations participate in the same value chain. This interaction creates trust between organizations and ensures ongoing participation, information sharing, and technology maintenance [145].

Table 11 shows the enablers identified for the processing link. Traceability, as a distinctive feature of the adoption of Food Informatics technologies, can help agri-food chains comply with commercial protocols so that economic returns can be generated from implementing these types of technologies [135]. Traceability is challenging to implement in agri-chains due to the complexity of suppliers and buyers [9]. Aligned with traceability, another factor that enables the implementation of technologies arises since it influences the awareness and intention of users to adopt technologies and plays an essential role in customer satisfaction [135]. This factor is the lack of transparency. It can generate a loss of quality and safety, causing damage to people and businesses, so it is essential to apply these technologies to products that can be mixed with others and generate cross-contamination [9].

These elements mentioned above allow for the implementation of technologies such as smart contracts that support the production, processing, and marketing links. Smart contracts facilitate disintermediation, reduce transaction costs and information asymmetry, ensure business transactions, and enable audit processes [135]. Implementing technologies such as IIoT and Blockchain, among others, promotes disintermediation by facilitating person-to-person operations through smart contracts. This reduces transaction costs, facilitates auditability and data protection, and eliminates intermediation nodes [143].

The participation of management is reflected in the orientation towards innovation and the organizational culture that can support the implementation of this type of technology [135]. The focus on innovation and culture directly impacts employees who feel confident in adopting and using this type of technology. Management is responsible for promoting strategies for the challenges that may emerge in the implementation processes [137]. Along these lines, when the digital strategy is well defined and aligned with the objectives and the processing and modernization of processes, the adoption processes are facilitated because of a clear commitment and vision [137]. This adoption allows for interconnection between the business units of the organization and the processing processes.

Ease of use anticipates the possibility of adopting Food Informatics technologies, especially when associated with aspects such as the cost of acquiring the technology [134]. Conversely, the reduction in labor intensity associated with adopting a technology [147], and that is directly perceived by employees, also plays a key role in adopting technologies, especially those of industry 4.0, such as robots and IIoT. These technologies can optimize production processes, reducing costs and eliminating waste. This reduction in costs, time, and waste has also been analyzed in the literature, especially through the implementation of robots in food manufacturing, because they eliminate the associated costs of training and educating employees, increase efficiency in using materials, and eliminate residues and waste. Finally, applying robots in the processes increases production speed, eliminates delays, fatigue, and absence due to illness, and increases productivity [7].

Implementing Food Informatics technologies in processing processes increases food quality and safety because errors derived from fatigue are reduced, control processes are made more efficient, and sensors and inspection systems ensure maximum quality [7]. The above, added to hygienic packaging, is essential to maintaining food safety because it helps keep food free from damage caused by environmental changes [149]. Eliminating human contact with food reduces incidents of cross-contamination, increasing compliance with legislation and food safety [7].

Consumers are increasingly aware of the importance of food quality and safety and the effects derived from consumption, so the environmental and social impacts derived from its production are also relevant [9]. Likewise, suppliers and competitors also generate an effect on the implementation of technologies such as Blockchain because if it is a standard already implemented in later links, requirements will be generated for its adoption to be accelerated and, thus, achieve its use and integration [9].

The regulations implemented by some countries and the variations in standards prevent full compliance with the requirements; some still maintain bar codes, others identification tags, and others traceability systems, so it is necessary to have global standards that promote compliance [9]. At the regulatory level, intellectual property, privacy, and copyright elements also become sources of protection that must be clearly defined by the responsible entities [141].

### 4.5. Barriers and Enablers Identified for the Marketing Link

Table 12 shows the barriers identified for the marketing link. In this link, support for technologies such as Blockchain, IoT, and RFID has been provided, aiming to facilitate the traceability of food “from farm to table” in an integrated, efficient, and transparent way [150]. Including this type of technology increases the demand for products at fair prices through digital models such as e-commerce and mobile applications, reducing intermediation [150]. Authors such as Liu et al. (2022) [151] have identified how the use of 4.0 technologies reduces the perception of risk by the final consumer. Likewise, if consumers are aware of the quality and safety of food, price volatility can be reduced by suppliers. In this sense, using this type of technology can help improve product prices and avoid their volatility. Several studies have explored applying flexible sensing technologies for food inspection [152,153]. These technologies, including visual, acoustic, chemical, and electronic sensors, enable the monitoring of product quality, safety, and freshness throughout the cold chain [152,153]. Advanced data analysis techniques, such as machine learning, deep learning, and neural networks, have further enhanced the capabilities of these detection methods, improving quality control and making progress in Food Informatics.

Other research approaches have analyzed how Food Informatics technologies contribute to reducing waste, especially related to confusion with expiration dates, over-purchasing, and inadequate purchasing plans [154]. This type of approach allows the chain to be integrated from front to back so that marketers of agri-food products can use this type of data, along with loyalty programs and the tracking of purchasing activities and recipes, among others [154].

**Table 12:** Barriers identified for this marketing link.

Barrier	Description	Technology	Authors
Data privacy and security	The privacy and security of information captured through various means of consumer preference or consumption patterns or preferences has an impact.	Blockchain	[6,155,156]
Data infrastructure and storage costs	The costs associated with deploying the infrastructure and its operation limit the adoption processes when these are high, and there are no subsidies or benefits.	Blockchain	[155,157]
Data infrastructure	The costs of servers and energy consumption to carry out the processes of capturing, storing, and processing information have an impact.	Blockchain	[155,157]
Lack of expertise	Technologies 4.0 require expertise not only to create them, but also to operate them. The above generates discomfort among operators.	Blockchain	[150,155]
Low innovation and entrepreneurship	The willingness to adopt technology and innovate also depends on the ability to recognize the value of information in the environment to apply it to their benefit.	Blockchain	[155]
Proven commercial viability	Technologies that have been validated under the normal conditions of use and operation and through pilots are more likely to be adopted because users can check the experiences of third parties.	Blockchain	[155]
Lack of trust between stakeholders	The lack of trust between participants in delivering information and maintaining its custody and integrity limits the exchange of data and the adoption of technologies.	Blockchain	[53]
Scalability	If the number of information capture nodes is low, the speed and number of transactions are limited.	Blockchain	[157]
Job losses	Using technologies that incorporate AI is considered a threat to job loss.	Artificial intelligence	[41]

Legislation aimed at data protection, privacy, and metadata monitoring, associated with national and international standards, requires the development of minimally acceptable international parameters for integrating data chains at a transnational level [155]. These elements have been associated in the literature with the lack of trust between stakeholders because data capture requires cooperation at all levels to avoid alterations and under-reporting [53]. Due to the increase in the transactions and users of IoT, QR, and Blockchain technologies, scalability is affected by the number of nodes required for simultaneous data processing [53].

The increase in infrastructure costs and service requirements is a limitation, even more so when small companies in the food sector decide to apply these technologies, which may subsequently present problems in their operation [53,155]. Data infrastructure is another element that adds to the costs because deploying technologies associated with the marketing link requires large investments for their capture, storage, and processing that, in many cases, are not weighed against value aggregation [155]. Finally, data storage costs: The data and metadata communicated through technologies such as Blockchain are not stored in one place. This implies that they are distributed in databases that are not found on a single server [53]. In addition, the amount of data required to achieve the extraction of knowledge and insights is high [41].

The lack of necessary technical knowledge and skills of the participants throughout the value chain, i.e., the low understanding of the technologies, can reduce motivation regarding implementation in the food sector. This is why training programs must be oriented on the use and potential relevance of technologies in different sectors of the economy [155]. Robotization and the use of artificial intelligence open the door to replacing personnel in some tasks, which is why some companies identify fear in employees adopting technologies due to the possibility of job loss or future replacement.

Finally, the low orientation towards innovation and entrepreneurship generates stagnation and delays in adopting technological innovations, leading to technological obsolescence, making the adoption of technologies much more complex and expensive since they imply much more radical changes [147].

Table 13 shows the enablers identified for the marketing link. For the marketing link, ease of use has been analyzed under four main criteria: how easy it is to learn about the technology, how easy it is to implement it, how easy it is to master it at the skill level, and how easy it is to use a complete system based on Food Informatics technology. This ease of use is mediated by attitude through the perceived usefulness of the technology [158].

Transparency is a characteristic valued by the final consumer, who assumes that the brand and the product they consume respects its value promise and for which they are willing to pay more, as it ensures that the information generated by the product has not been manipulated [150]. This transparency is complemented by traceability, which facilitates the tracking and identification of processes developed with the food to be consumed. Hence, it can be developed through QR codes, reducing fake and illegal products in the market and increasing customer confidence [53].

Social prestige has been analyzed in the literature as one of the most important attributes when making decisions to adopt technologies [159], finding that the positioning of a brand or a product influences its adoption process [149]. Likewise, the perceived quality of the product is mediated by traceability, that is, the use of Food Informatics technologies in the traceability of food directly influences the perception and purchase of the product by the consumer [52]. Similarly, the literature shows that the use of labels associated with Blockchain traceability increases the perception of quality on those products that use other types of visual elements, such as QR codes, making the purchase intention increase, especially in unfamiliar brands [52].

The reduction of transaction costs has been identified as an enabler of the adoption of Blockchain technologies because logistics and transportation costs can be reduced, allowing some taxes to be avoided [150]. Auditability is mainly associated with the responsibility of the parties to keep records immutable and accessible to potential verifiers because a slight change in a record could be identified as a fraud that affects the entire production chain [150].

Domestic policies enable and motivate the adoption of Food Informatics technologies. However, the contrary occurs if these are not clear in their implementation and support or if, on the contrary, the technologies that these policies provide are not commercially validated and are offered as solutions to problems presented in the marketing link that are not replicable in all chains due to their conditions, particular specifications, or both [156].

## 5. Discussion

The complex panorama of agri-food chains poses a set of technological, social, economic, organizational, and institutional factors for effectively developing Food Informatics technology adoption processes. This literature review addressed the barriers and enablers for the adoption of Food Informatics technologies from a global view of the agri-food chain, understanding that there are gaps that have not yet been analyzed to seek a real integration of the links not only from the productive, but from the technological point of view to achieve two-way communication between client/market and producer, without leaving aside the importance of the processing link as a source of value aggregation and alignment with the real demands of the market.

**Figure 9:**
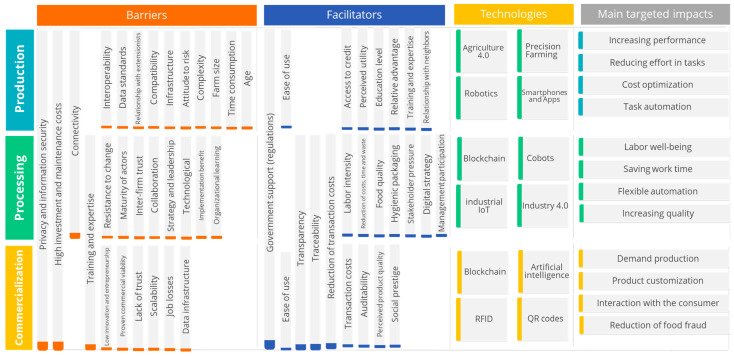
Cross-analysis of barriers and enablers or facilitators for the adoption of Food Informatics technologies in agri-food chains.

By carrying out a joint analysis of the barriers and enablers for the adoption of Food Informatics technologies in agri-food chains, it is evident that there are analogous barriers between the three links analyzed, such as data privacy and security, which becomes a determining factor for the deployment of data ecosystems that require the permanent uploading of information, its legitimacy, and its immutability (see Figure 9). Therefore, knowing clearly where the data will be stored, who can access it, and the security guarantees becomes a vital element in facilitating the participation of the various parties. On the other hand, there are the investment and maintenance costs of this type of technology, which mostly require particular infrastructure for their correct deployment, such as the Internet and electricity, in addition to having trained personnel for their correct operation. Internet connectivity is also a barrier evident in the first two links of the chain, especially in production, because there is no 100% coverage in rural areas, and concerning training and expertise, these are focused on the processing and marketing links.

The other barriers identified are specific to each link based on its particular requirements. In the production link, farm location is seen as a factor influencing the choice of whether to adopt these technologies or not. Farms in mountainous areas are less likely to adopt technologies than others in flat areas [112]. These findings may be interesting, especially from the perspective of adopting Food Informatics technologies in countries such as Colombia, which has a mountainous geography, and particularly for products such as coffee and cocoa, where the levels of technological adoption are much lower [98]. In contrast, flat-area crops such as rice have a much greater capacity for technological adoption [116]. Similarly, other studies have been aimed at identifying differences in adoption patterns based on the geographical location of the farms [83,98]. For the production link, the main predictors are associated with benefits for crop management, increased production, and reduced costs [10]. Another relevant aspect is the recommendability of other users, which improves usability and allows for a better understanding of the usefulness of the technology [10]. An interesting element for the production link is how the belief in reducing the environmental impact derived from implementing Food Informatics technologies can facilitate the adoption process [10,79,104,115].

For the marketing link, a critical barrier to the adoption of technologies is associated with organizational innocence seen from the leadership and the strategy that the company deploys to facilitate the adoption processes and improve its performance, that is, if management is clear about the benefits and use of the technology, its adoption is facilitated. Other research suggests that the lack of awareness about the potential benefits also becomes a critical barrier to implementing supply chain 4.0 [140]. Studies show the importance of developing technological competencies and their homogeneity when implementing or adopting Food Informatics technologies in agri-food chains. This level of maturity emerges as a key driver at the time of introduction into the implementation processes [144].

In the marketing link, research shows that the willingness to share data across the value chain using Food Informatics technologies implies a high level of commitment and responsibility that must be compensated by profits or value on the part of the consumer to generate a return on that effort [137]. The easiest way for consumers to access information is by scanning a QR code. Different studies agree that having information available in this way helps increase trust and transparency among customers in chains such as coffee, meat, vegetables, eggs, and milk [53]. However, at the trust and transparency levels, other studies have shown that seals associated with Blockchain traceability increase the perceived quality of the products, positively impacting their purchase intention, compared to the use of QR seals that do not have the same effect on traditional brands or brands positioned in the market [52].

Another important aspect is the scalability of this type of technology, as indicated by Vu et al. [9]. These authors stated that many of the Food Informatics technology implementation initiatives are still considered pilots or small-scale projects, which is why it becomes a significant barrier from the perspective of the integration of the links in a business model that benefits all those involved. In turn, public policy incentives or regulations could be decisive in facilitating these types of transactions [9].

This study reveals that age and educational level significantly influence the perceived complexity of adopting Food Informatics technologies. While certain barriers can act as enablers, such as a higher level of education, it is clear that advanced technologies may require specialized training to improve their usability. Research indicates that postgraduate-level farmers are likelier to adopt precision agriculture technologies, especially at the master’s level.

For the processing link, the maturity and preparation of chain actors are crucial factors for adoption. Supportive infrastructure, human capital, and organizational support can facilitate the implementation of Food Informatics technologies. Conversely, the combined costs, maintenance, and infrastructure barriers for the marketing link can hinder adoption. To address this, policies promoting financing and accessibility to these technologies are essential to boost adoption rates.

### Research Implications

The main practical implications of this study are the identification of enablers analogous to the three links that could promote the adoption of this type of technology, such as government support and regulations. These are considered the main drivers because they create the enabling conditions for the promotion of technologies and facilitate their adoption by the actors in each link. Likewise, ease of use is concentrated in the production and marketing links because the complexity of the technologies limits their adoption (see Figure 9).

Transparency, traceability, and reduction of transaction costs appear as enablers or facilitators in the marketing and processing links. Regarding the first two, the literature shows them as the main motivators for adopting technologies because they increase consumer confidence in the products that reach the market. Furthermore, they have higher quality and safety derived from using automation technologies, in addition to the verification that can be conducted through Blockchain. For transaction costs, these links also consider that Food Informatics technologies can reduce the logistics costs associated with the trade of agri-food products, bringing the end customer closer to the links mentioned above.

While training and expertise are barriers in the processing and marketing links, training and expertise are enablers or facilitators in the production link. This is particularly relevant mainly due to the complexity of the technologies deployed and the greater infrastructure required in these links. In addition to examining adoption determinants, this study offers a bibliometric analysis of the evolving scientific interest in Food Informatics technologies. The analysis explores the global research landscape, focusing on institutions and researchers that have incorporated these technologies into agri-food systems. The study highlights the growing recognition of Food Informatics as a valuable tool for enhancing food chain performance.

## 6. Conclusions

The identified factors are linked to each other through systemic interactions, which implies that they are independent of each other and that one factor can generate negative or positive changes in others, so they cannot be understood individually [160]. These are, in general terms, social (ease of use, transparency, and traceability), economic (initial investment costs, return on investment, and access to credit), and technological (data privacy and connectivity) factors [81].

The implementation of Food Informatics technologies requires the participation of a large number of nodes for data capture. However, if the nodes are small firms or small producers with limited technological expertise and scarce financial resources, it could become a limitation to keep the system operational and the flow of information managed for all participants [9]. Studies show that the primary motivations for adopting food informatics technologies are associated with the desire to obtain information to improve crop management [103], the optimization of processing processes, and the increase in demand for agri-food products. In some of the cases studied, it is considered necessary that the information linked to Food Informatics processes be previously validated by certifying bodies to protect all parties, especially the final consumer [136].

The literature shows that there is a direct relationship between the implementation of Food Informatics technologies (Blockchain) and compliance with voluntary standards in some chains such as cocoa and coffee, as is the case in “Choco4Peace” (more information at https://www.choco4peace.com/, accessed on 24 July 2024), which allows vulnerable Colombian farmers to improve their lives by finding markets for their cocoa, allowing them to escape poverty and conflict [161]. In this sense, Choco4peace measures the economic, social, and environmental benefits of the investments developed based on the data recorded in Blockchain.

Adopting technologies such as Agriculture 4.0, precision agriculture, Blockchain, robotics, IoT, and artificial intelligence in food chains requires new roles, understanding, and skills necessary to support all aspects of the technology, which generates a barrier to its adoption [162]. In general terms, to achieve an efficient adoption of Food Informatics technologies, the following are required: (1) Achieve a change in mentality, culture, and skills that facilitates the integration and adoption of technologies; (2) generate public policies that allow access to energy and connectivity infrastructure, especially in remote areas, to achieve adoption processes; (3) integrate academia through research, technology development, and innovation processes for the promotion of technologies and the generation of capacities from the territory; and finally, (4) use data ecosystem models that connect the three links (production, processing, and commercialization) through effective elements of data governance, cybersecurity that facilitates transparency, and the use of data by the parties involved.

A key limitation of this study was the independent analysis of barriers and enablers to Food Informatics adoption in agri-food chains. While the literature suggests interdependencies among these factors, this study treated them separately. This study recommends that future research develop a computational model that simulates the adoption process to address this issue. This model, incorporating hierarchical structures, would allow for a more nuanced understanding of how these factors interact, influencing each other’s impact on adoption. Such a model could provide decision-makers with more refined policy guidelines for promoting Food Informatics adoption in agri-food chains.

## Figures and Tables

**Table 2:** Search criteria and keywords defined for the cultivation link.

Keywords Associated with the Use of Technologies in Cultivation	Keywords Associated with the Cultivation Link in Agri-Food Chains	Keywords Associated with Adoption and Use	Keywords Associated with Decision Factors
Smart farming Smart monitoring Precision farming Agriculture 4.0 Digital* agriculture* Precision Agriculture	Crop Farm Agriculture Cultivation	Adoption Appropriation Transfer Acquisition Implementation Use Application	determinant* inhibitor* driver* enabler* barrier* “Influential Elements” motiva*

**Table 3:** Search criteria and keywords defined for the processing link.

Keywords Associated with the Use of Technologies in Processing (Transformation)	Keywords Associated with the Processing Link in Agri-Food Chains	Keywords Associated with Adoption and Use	Keywords Associated with Decision Factors
IiOt Industry 4.0 “Industrial IoT” Smart Factory Smart Manufacturing Industrial robot Blockchain Industrial Internet of Things	“Food manufacturing” “Food processing” “Food industr*” Food science Food Supply Chain	Adoption Appropriation Transfer Acquisition Implementation Use	determinant* inhibitor* driver* enabler* barrier* “Influential Elements” motiva*

**Table 4:** Search criteria and keywords defined for the marketing link.

Keywords Associated with the Use of Technologies in Marketing	Keywords Associated with the Marketing Link in Agri-Food Chains	Keywords Associated with Adoption and Use	Keywords Associated with Decision Factors
Food computing Internet of food Food Informatics Augmented Reality Blockchain Computer Vision Machine Learning Deep Learning Sensor Technology Mobile Applications	Food Quality Food Storage Food Packaging Food Products “Food branding” “Food advertising” “Food promotion” Food Preference Food Labeling Food Consumption Food recommendation Food design	Adoption Appropriation Transfer Acquisition Implementation Use application	determinant* inhibitor* driver* enabler* barrier* “Influential Elements” motiva*

**Table 5:** Query algorithm and the number of results for each database used for the cultivation link.

Query Algorithm	# of Results
Scopus (((TITLE-ABS-KEY (“crop”)) OR (TITLE-ABS-KEY (“farm*”)) OR (TITLE-ABS-KEY (“agriculture”)) OR (TITLE-ABS-KEY (“cultivation”))) AND (TITLE-ABS-KEY (“smart monitoring”) OR TITLE-ABS-KEY (“Smart Farming”) OR TITLE-ABS-KEY (“Precision Agriculture”) OR TITLE-ABS-KEY (“Precision farm*”) OR TITLE-ABS-KEY (“Agriculture 4.0”) OR TITLE-ABS-KEY (“Digital* agriculture*”) OR TITLE-ABS-KEY (“Precision Agriculture”)) AND ((TITLE-ABS-KEY (adoption)) OR (TITLE-ABS-KEY (appropriation)) OR (TITLE-ABS-KEY (transfer)) OR (TITLE-ABS-KEY (acquisition)) OR (TITLE-ABS-KEY (implementation)) OR (TITLE-ABS-KEY (use)) OR (TITLE-ABS-KEY (Application))) AND (TITLE-ABS-KEY (determinant*) OR TITLE-ABS-KEY (inhibitor*) OR TITLE-ABS-KEY (driver*) OR TITLE-ABS-KEY (enabler*) OR TITLE-ABS-KEY (motiva*) OR TITLE-ABS-KEY (barrier*) OR TITLE-ABS-KEY (“Influential Elements”))) PUBYEAR > 2013	191
WoS (((TI = crop OR AB = crop OR AK = crop) OR (TI = farm* OR AB = farm* OR AK = farm*) OR (TI = agriculture OR AB = agriculture OR AK = agriculture) OR (TI = cultivation OR AB = cultivation OR AK = cultivation)) AND ((TI = “Smart farming” OR AB = “Smart farming” OR AK = “Smart farming”) OR (TI = “smart monitoring” OR AB = “smart monitoring” OR AK = “smart monitoring”) OR (TI = “Precision farming” OR AB = “Precision farming” OR AK = “Precision farming”) OR (TI = “Agriculture 4.0” OR AB = “Agriculture 4.0” OR AK = “Agriculture 4.0”) OR (TI = “Digital* agriculture*” OR AB = “Digital* agriculture*” OR AK = “Digital* agriculture*”) OR (TI = “Precision Agriculture” OR AB = “Precision Agriculture” OR AK = “Precision Agriculture”)) AND ((TI = Adoption OR AB = Adoption OR AK = Adoption) OR (TI = Appropriation OR AB = Appropriation OR AK = Appropriation) OR (TI = Transfer OR AB = Transfer OR AK = Transfer) OR (TI = Acquisition OR AB = Acquisition OR AK = Acquisition) OR (TI = Implementation OR AB = Implementation OR AK = Implementation) OR (TI = Use OR AB = Use OR AK = Use) OR (TI = Application OR AB = Application OR AK = Application)) AND ((TI = determinant* OR AB = determinant* OR AK = determinant*) OR (TI = inhibitor* OR AB = inhibitor* OR AK = inhibitor*) OR (TI = driver* OR AB = driver* OR AK = driver*) OR (TI = enabler* OR AB = enabler* OR AK = enabler*) OR (TI = barrier* OR AB = barrier* OR AK = barrier*) OR (TI = “Influential Elements” OR AB = “Influential Elements” OR AK = “Influential Elements”) OR (TI = motiva* OR AB = motiva* OR AK = motiva*))) AND PY = (2014–2024)	271

**Table 6:** Query algorithm and the number of results for each database used for the processing link.

Query Algorithm	# of Results
Scopus ((TITLE-ABS-KEY (“food manufactu*”) OR TITLE-ABS-KEY (“food proces*”) OR TITLE-ABS-KEY (“food industr*”) OR TITLE-ABS-KEY (“food science”) OR TITLE-ABS-KEY (“food supply chain”))) AND ((TITLE-ABS-KEY (“iiot”) OR TITLE-ABS-KEY (“industry 4.0”) OR TITLE-ABS-KEY (“industrial iot”) OR TITLE-ABS-KEY (“smart factory”) OR TITLE-ABS-KEY (“smart manufact*”) OR TITLE-ABS-KEY (“industrial robot”) OR TITLE-ABS-KEY (blockchain) OR TITLE-ABS-KEY (“industrial internet of thing*”))) AND ((TITLE-ABS-KEY (adoption) OR TITLE-ABS-KEY (appropriation) OR TITLE-ABS-KEY (transfer) OR TITLE-ABS-KEY (acquisition) OR TITLE-ABS-KEY (implementation) OR TITLE-ABS-KEY (use))) AND ((TITLE-ABS-KEY (determinant*) OR TITLE-ABS-KEY (inhibitor*) OR TITLE-ABS-KEY (driver*) OR TITLE-ABS-KEY (enabler*) OR TITLE-ABS-KEY (motiva*) OR TITLE-ABS-KEY (barrier*) OR TITLE-ABS-KEY (“influential element*”))) PUBYEAR > 2013	42
WoS ((TI = IiOt OR AB = IiOt OR AK = IiOt) OR (TI = “Industry 4.0” OR AB = “Industry 4.0” OR AK = “Industry 4.0”) OR (TI = “Smart Factory” OR AB = “Smart Factory” OR AK = “Smart Factory”) OR (TI = “Smart Manufacturing” OR AB = “Smart Manufacturing” OR AK = “Smart Manufacturing”) OR (TI = “industrial robot” OR AB = “industrial robot” OR AK = “industrial robot”) OR (TI = Blockchain OR AB = Blockchain OR AK = Blockchain) OR (TI = “Industrial Internet of Things” OR AB = “Industrial Internet of Things” OR AK = “Industrial Internet of Things”)) AND ((TI = “Food manufacturing” OR AB = “Food manufacturing” OR AK = “Food manufacturing”) OR (TI = “food processing” OR AB = “food processing” OR AK = “food processing”) OR (TI = “Food industr*” OR AB = “Food industr*” OR AK = “Food industr*”) OR (TI = “Food science” OR AB = “Food science” OR AK = “Food science”) OR (TI = “Food Supply Chain” OR AB = “Food Supply Chain” OR AK = “Food Supply Chain”)) AND ((TI = Adoption OR AB = Adoption OR AK = Adoption) OR (TI = Appropriation OR AB = Appropriation OR AK = Appropriation) OR (TI = Transfer OR AB = Transfer OR AK = Transfer) OR (TI = Acquisition OR AB = Acquisition OR AK = Acquisition) OR (TI = Implementation OR AB = Implementation OR AK = Implementation) OR (TI = Use OR AB = Use OR AK = Use) OR (TI = Application OR AB = Application OR AK = Application)) AND ((TI = determinant* OR AB = determinant* OR AK = determinant*) OR (TI = inhibitor* OR AB = inhibitor* OR AK = inhibitor*) OR (TI = driver* OR AB = driver* OR AK = driver*) OR (TI = enabler* OR AB = enabler* OR AK = enabler*) OR (TI = barrier* OR AB = barrier* OR AK = barrier*) OR (TI = “Influential Elements” OR AB = “Influential Elements” OR AK = “Influential Elements”) OR (TI = motiva* OR AB = motiva* OR AK = motiva*)) AND PY = (2014–2024)	48

**Table 7:** Query algorithm and the number of results for each database used for the marketing link.

Query Algorithm	# of Results
Scopus ((TITLE-ABS-KEY (“Food branding”) OR TITLE-ABS-KEY (“Food advertising”) OR TITLE-ABS-KEY (“Food promotion”) OR TITLE-ABS-KEY (“food marketing”) OR TITLE-ABS-KEY (“Food Preference”) OR TITLE-ABS-KEY (“Food Labeling”) OR TITLE-ABS-KEY (“Food Consumption”) OR TITLE-ABS-KEY (“Food recommendation”) OR TITLE-ABS-KEY (“Food Quality”) OR TITLE-ABS-KEY (“Food Storage”) OR TITLE-ABS-KEY (“Food Packaging”) OR TITLE-ABS-KEY (“Food Products”) OR TITLE-ABS-KEY (“food design”))) AND ((TITLE-ABS-KEY (“Food computing”) OR TITLE-ABS-KEY (“Internet of food”) OR TITLE-ABS-KEY (“Food informatic*”) OR TITLE-ABS-KEY (“Augmented Reality”) OR TITLE-ABS-KEY (blockchain) OR TITLE-ABS-KEY (“Computer Vision”) OR TITLE-ABS-KEY (“Machine Learning”) OR TITLE-ABS-KEY (“Deep Learning”) OR TITLE-ABS-KEY (“Sensor Technology”) OR TITLE-ABS-KEY (“Mobile Application*”))) AND ((TITLE-ABS-KEY (adoption) OR TITLE-ABS-KEY (appropriation) OR TITLE-ABS-KEY (transfer) OR TITLE-ABS-KEY (acquisition) OR TITLE-ABS-KEY (implementation) OR TITLE-ABS-KEY (use) OR TITLE-ABS-KEY (application))) AND ((TITLE-ABS-KEY (determinant*) OR TITLE-ABS-KEY (inhibitor*) OR TITLE-ABS-KEY (driver*) OR TITLE-ABS-KEY (enabler*) OR TITLE-ABS-KEY (motiva*) OR TITLE-ABS-KEY (barrier*) OR TITLE-ABS-KEY (“influential element*”))) PUBYEAR > 2013	15
WoS (((TI = “Food computing” OR AB = “Food computing” OR AK = “Food computing”) OR (TI = “Internet of food” OR AB = “Internet of food” OR AK = “Internet of food”) OR (TI = “Food informatics” OR AB = “Food informatics” OR AK = “Food informatics”) OR (TI = “Augmented Reality” OR AB = “Augmented Reality” OR AK = “Augmented Reality”) OR (TI = Blockchain OR AB = Blockchain OR AK = Blockchain) OR (TI = “Computer Vision” OR AB = “Computer Vision” OR AK = “Computer Vision”) OR (TI = “Machine Learning” OR AB = “Machine Learning” OR AK = “Machine Learning”) OR (TI = “Deep Learning” OR AB = “Deep Learning” OR AK = “Deep Learning”) OR (TI = “Sensor Technology” OR AB = “Sensor Technology” OR AK = “Sensor Technology”) OR (TI = “Mobile Application*” OR AB = “Mobile Application*” OR AK = “Mobile Application*”)) AND ((TI = “Food Products” OR AB = “Food Products” OR AK = “Food Products”) OR (TI = “Food branding” OR AB = “Food branding” OR AK = “Food branding”) OR (TI = “Food advertising” OR AB = “Food advertising” OR AK = “Food advertising”) OR (TI = “Food promotion” OR AB = “Food promotion” OR AK = “Food promotion”) OR (TI = “Food Preference” OR AB = “Food Preference” OR AK = “Food Preference”) OR (TI = “Food Labeling” OR AB = “Food Labeling” OR AK = “Food Labeling”) OR (TI = “Food Consumption” OR AB = “Food Consumption” OR AK = “Food Consumption”) OR (TI = “Food recommendation” OR AB = “Food recommendation” OR AK = “Food recommendation”) OR (TI = “food design” OR AB = “food design” OR AK = “food design”)) AND ((TI = Adoption OR AB = Adoption OR AK = Adoption) OR (TI = Appropriation OR AB = Appropriation OR AK = Appropriation) OR (TI = Transfer OR AB = Transfer OR AK = Transfer) OR (TI = Acquisition OR AB = Acquisition OR AK = Acquisition) OR (TI = Implementation OR AB = Implementation OR AK = Implementation) OR (TI = Use OR AB = Use OR AK = Use) OR (TI = Application OR AB = Application OR AK = Application)) AND ((TI = determinant* OR AB = determinant* OR AK = determinant*) OR (TI = inhibitor* OR AB = inhibitor* OR AK = inhibitor*) OR (TI = driver* OR AB = driver* OR AK = driver*) OR (TI = enabler* OR AB = enabler* OR AK = enabler*) OR (TI = barrier* OR AB = barrier* OR AK = barrier*) OR (TI = “Influential Elements” OR AB = “Influential Elements” OR AK = “Influential Elements”) OR (TI = motiva* OR AB = motiva* OR AK = motiva*))) AND PY = (2014–2024)	26

**Table 10:** Barriers identified for the processing link.

Barrier	Description	Chain	Technology	Reference
Connectivity	Access to energy and Internet infrastructure is key to deploying technologies associated with real-time data capture.	Wine	Blockchain	[133]
Governance, privacy, and information security	Data privacy related to the different links presents a risk for the associated parties.	Fruits	Blockchain	[133,134,135]
Training and expertise of collaborators	Digital skills are necessary for the implementation of technologies.	Wine Processing of fruits and vegetables	Blockchain Robotization	[9,133,136,137,138,139]
Collaboration	The joint development of actions for the implementation of technologies improves the performance of the process.	Wine Cotton Food and beverage industry	Blockchain Industry 4.0	[136,138,140]
Technological	Industrial equipment does not guarantee the standardization of products and, if necessary, is obsolete.	Bakery	Industry 4.0	[38]
Organizational learning	Employee skills, educational level, and absorption capacity have an impact.	Not mentioned	Blockchain	[141]
Strategy and leadership	Integrated knowledge management strategies, clear purposes, strategic planning, and relationships have an impact.	Not mentioned	Blockchain	[141]
High investment and maintenance costs	Initial investments are required to implement Food Informatics solutions, and their implementation does not guarantee immediate return.	Wine Processing of fruits and vegetables Cotton	Blockchain Industry 4.0	[9,133,135,137,138,142,143]
Level of preparation or maturity of the actors in the chain	The level of preparation of the partners is derived from the social influence raised by the UTAUT model.	Not mentioned	Blockchain	[144]
Lack of knowledge of the exact benefits of implementation	The lack of knowledge of the cost–benefit relationship of implementing new technologies results in uncertainty that is difficult to assume.	Food and beverage industry	Industry 4.0	[140]
Resistance to change	Resistance on the part of organizations and employees during the adoption process has an impact.	Fruits	Blockchain	[135]
Inter-firm trust	It is an essential factor when several organizations are present in the same value chain.	Non mentioned	Blockchain	[145]

**Table 11:** Enablers identified for the processing link.

Enabler	Description	Chain	Technology	Reference
Transparency	It ensures quality and information, precision, and credibility towards the market.	Not mentioned	Blockchain	[9,134,145]
Traceability	It facilitates the real-time tracking of products (quantities, origins, and destination dates).	Wine	Blockchain	[9,134,143]
Management involvement	It guarantees funds and resources to participate in the adoption processes.	Not mentioned	Not mentioned	[146]
Digital strategy	Agri-businesses should have a clear digital processing (transformation) strategy to promote the use and implementation of technology.	Fruits processing	Robotization	[137]
Policies and regulations	Clear regulations and policies are required at the national level to adopt technologies.	Cotton	Industry 4.0	[138]
Reduction of labor intensity	When the perception of the benefit of technology is in reducing the workload, it can drive the adoption of technology.	Not mentioned	Industry 4.0	[147]
Reduction of transaction costs	The elimination of intermediaries reduces transaction costs.	Wine	Blockchain	[143]
Reduction of costs, time, and waste	The implementation of technologies eliminates the costs associated with employee training, reduces production times by increasing productivity, and also increases the efficient use of materials.	Poultry Bovine	Robotization Blockchain and IoT	[7,69,148]
Food quality and safety	Quality and safety are vital parameters for the final consumer, so their assurance will reduce fraud and the lack of standardization.	Not mentioned	Blockchain and IoT	[148]
Hygienic packaging	The increase in control points directly impacts traceability in the hygiene of handling processes.	Not mentioned	Blockchain and IoT	[148]
Pressure from buyers/suppliers, competitors, and consumers	The links in logistics, marketing, and consumption indirectly pressure the implementation of technologies to link them to their own systems.	Not mentioned	Blockchain	[9]

**Table 13:** Enablers identified for the marketing link.

Enabler	Description	Chain	Technology	Authors
Social prestige	Depending on the positioning of the brand or product, customers may be more willing to participate in the adoption and use of technology processes.	Fresh products	Blockchain–RFID	[149]
Transparency	Transparency is associated with the ability to track the journey of a product from the production link to the consumer, generating trust.	Not mentioned	Blockchain–RFID	[150]
Traceability	Traceability allows for the tracking of products throughout the value chain through shared and immutable records.	Not mentioned	Blockchain–RFID	[150]
Auditability	Linking stakeholders in the model allows for the auditability of the parties.	Not mentioned	Blockchain–RFID	[150]
Reduction of transaction costs	Technologies 4.0 help reduce logistics costs and related taxes.	Not mentioned	Blockchain–RFID	[150,156]
Ease of use	If the system is easy to implement and use, it allows marketers to implement them in their businesses.	Not mentioned	Blockchain	[158]
Perceived quality of the product	The information available through Food Informatics technologies affects the purchase intention.	Not mentioned	Blockchain	[52]
Government support (regulations)	Local and national policies based on solving real bottlenecks, compared to others that encourage the adoption of specific technologies, can be effective.	Not mentioned	Blockchain	[53,156]

## Data Availability

No new data were created or analyzed in this study. Data sharing is not applicable to this article.
